# Contemporary Update on Clinical and Experimental Prostate Cancer Biomarkers: A Multi-Omics-Focused Approach to Detection and Risk Stratification

**DOI:** 10.3390/biology13100762

**Published:** 2024-09-25

**Authors:** Sana Hachem, Amani Yehya, Jad El Masri, Nicole Mavingire, Jabril R. Johnson, Abdulrahman M. Dwead, Naim Kattour, Yazan Bouchi, Firas Kobeissy, Soroush Rais-Bahrami, Yehia Mechref, Wassim Abou-Kheir, Leanne Woods-Burnham

**Affiliations:** 1 Department of Anatomy, Cell Biology, and Physiological Sciences, American University of Beirut, Beirut 1107-2020, Lebanonay46@aub.edu.lb (A.Y.);; 2 Department of Surgery, Morehouse School of Medicine, Atlanta, GA 30310, USA; nmavingire@msm.edu (N.M.);; 3 Department of Microbiology, Biochemistry, & Immunology, Morehouse School of Medicine, Atlanta, GA 30310, USA; jabjohnson@msm.edu; 4 Department of Neurobiology, Morehouse School of Medicine, Atlanta, GA 30310, USA; 5 Department of Urology, Heersink School of Medicine, University of Alabama at Birmingham, Birmingham, AL 35294, USA; 6 Department of Radiology, Heersink School of Medicine, University of Alabama at Birmingham, Birmingham, AL 35294, USA; 7 O’Neal Comprehensive Cancer Center, Heersink School of Medicine, University of Alabama at Birmingham, Birmingham, AL 35294, USA; 8 Department of Chemistry and Biochemistry, Texas Tech University, Lubbock, TX 79409, USA

**Keywords:** omics, multi-omics, prostate cancer, biomarkers

## Abstract

**Simple Summary:**

Multi-omics has become a crucial tool in cancer biomarker discovery over the last decade, including for prostate cancer. This approach integrates data from various biological layers, enhancing our understanding of complex, heterogeneous cancers. Multi-omics accelerates precision medicine by identifying new biomarkers and therapeutic targets, potentially improving treatment options for advanced prostate cancer patients. This review discusses traditional prostate cancer biomarkers and highlights recent single-omics and multi-omics advances that have improved clinical diagnostic and prognostic capabilities.

**Abstract:**

Prostate cancer remains a significant health challenge, being the most prevalent non-cutaneous cancer in men worldwide. This review discusses the critical advancements in biomarker discovery using single-omics and multi-omics approaches. Multi-omics, integrating genomic, transcriptomic, proteomic, metabolomic, and epigenomic data, offers a comprehensive understanding of the molecular heterogeneity of prostate cancer, leading to the identification of novel biomarkers and therapeutic targets. This holistic approach not only enhances the specificity and sensitivity of prostate cancer detection but also supports the development of personalized treatment strategies. Key studies highlighted include the identification of novel genes, genetic mutations, peptides, metabolites, and potential biomarkers through multi-omics analyses, which have shown promise in improving prostate cancer management. The integration of multi-omics in clinical practice can potentially revolutionize prostate cancer prognosis and treatment, paving the way for precision medicine. This review underscores the importance of continued research and the application of multi-omics to overcome current challenges in prostate cancer diagnosis and therapy.

## 1. Introduction

Prostate cancer (PC) ranks as the second most prevalent solid tumor cancer among men, and the most common non-cutaneous cancer in American men. In 2020, an estimated 1.4 million new cases of PC were diagnosed worldwide, accounting for 14.1% of new cancer cases in males [1]. The multifactorial disease has significant variation in morbidity and mortality incidence across a wide range of ethnic groups and different regions. Developed regions like North America and Northern and Western Europe, among others, have a notably higher PC prevalence when compared to less developed regions like Asia and Africa; this might be due to different factors like genetic predisposition and healthcare access [2]. PC incidence also varies by ethnicity, with Black men exhibiting the highest prevalence (24.7%) followed by white men (19.8%), and Asian men having the lowest PC incidence (13.4%) [3].

A multitude of somatic and germline mutations are involved in the complexity of the genetic composition of PC. Aberrations in tumor suppressor genes (e.g., TP53, PTEN, etc.), rearrangements of transcription genes like E26 transformation-specific (ETS) fusion, and changes in the androgen receptor (AR) signaling pathway are all important genetic alterations influencing PC progression [4]. In addition, PC prognosis can be affected by a myriad of different comorbidities such as obesity, diabetes, and cardiovascular disease, all of which can also affect disease management. Treatment choices and the ability to receive a curative treatment are also largely affected by chronological age [5]. This suggests that the intricate mechanisms of PC are influenced by a complex interplay of physiological, environmental, and genetic factors, characterizing it as a heterogeneous disease. Due to the multifactorial nature of the disease, clinical prognostic factors like grading via Gleason scoring and tumor, node, and metastasis (TNM) staging can only explain a portion of the clinical treatment responses experienced [6,7].

Inadequate diagnosis due to unnecessary biopsies can result in several negative repercussions, including severe infection, abscess formation, and fistula development, as well as potential long-term complications such as urinary retention and erectile dysfunction [8]. Additionally, patients may experience significant psychological burden due to procedure-related distress and side effects [9]. To identify diagnostic tools that better manage PC, biomarkers can serve as vital components to stratify and diagnose PC. Early detection of PC utilizes prostate-specific antigen (PSA) as a key biomarker. However, due to its limited specificity, more recent advances in novel diagnostic biomarkers have been pursued [10]. An enhanced predictive value and a more accurate diagnosis for PC have been suggested through the identification of novel biomarkers like prostate cancer antigen 3 (PCA3) and transmembrane protease serine 2 ETS-related gene (TMPRSS2-ERG) among several others [11]. The detection of additional biomarkers to incorporate into clinical practice represents a critical step toward improving personalized treatment strategies.

A multi-omics approach provides an extensive overview of the molecular landscape of PC and offers a revolutionary tactic in the field of biomarker discovery. The intricate mechanisms and relationships that drive PC and other cancers can be uncovered by analyzing genomic, proteomic, metabolomic, transcriptomic, and epigenomic datasets [11]. Integrating multidisciplinary information within a comprehensive and holistic framework holds excellent potential to identify new biomarkers and novel therapeutic targets that will pave the way for precision oncology. This review comprehensively highlights the capability of a multi-omics approach to improve our understanding of PC while shedding light on the remaining challenges and the future implications for clinical translation.

## 2. Traditional Biomarkers in Prostate Cancer

### 2.1. Protein Biomarkers—PSA

Annually in the United States, over 1.3 million men undergo biopsies for diagnosing suspected prostate malignancies, 85% of which will lack confirmation of clinically significant PC. The unfortunate result is that many men experience an invasive procedure that yields risks of procedural complications that may have been avoidable [12]. Prior to the biopsy, PSA is used as the gold standard, population-based screening tool. PSA testing has long been the cornerstone of PC screening, monitoring disease progression, and evaluating therapeutic responses [13]. However, PSA lacks specificity for PC as elevated levels can also result from benign conditions like prostatitis and benign prostatic hyperplasia (BPH). This often leads to false-positive results and unnecessary biopsies, contributing to patient anxiety, increased healthcare costs, and potential biopsy and treatment-related complications [14].

### 2.2. PSA Derivatives and Related Biomarkers

To improve the specificity of PSA testing, several derivatives and related biomarkers have been developed [13].

#### 2.2.1. Free PSA (fPSA)

The ratio of free to total PSA can help differentiate PC from benign prostate conditions in patients with PSA levels ranging from 4–10 ng/mL. This glycan-based biomarker offers a more precise assessment of PC risk, reducing the burden of unnecessary biopsies and improving patient outcomes. Recent advancements in fPSA glycoprofiling have significantly improved its diagnostic accuracy [15,16]. Analyzing the glycan structures on fPSA can enhance the discrimination between PC and non-cancerous prostatic conditions. This approach, known as the Prostate Glycan Index (PGI), showed a Receiver Operating Characteristic (ROC) analysis with an area under the curve (AUC) value of 0.821, outperforming the traditional fPSA and the Prostate Health Index (PHI) [15]. The implementation of PGI in the clinic could potentially avoid 63.5% of unnecessary biopsies, compared to 17.5% with fPSA and 33.3% with PHI, while maintaining high sensitivity and specificity.

#### 2.2.2. Prostate Health Index (PHI)

The PHI combines total PSA, fPSA, and [-2]proPSA (p2PSA) to provide a more accurate risk assessment for PC, especially within the PSA grey zone (4–10 ng/mL). Recent data have also demonstrated that PHI significantly improves predictive accuracy compared to PSA alone [17]. The modified Prostate Health Index (mPHI-2), which incorporates prostate volume and diameter measurements, outperformed traditional PHI and other PSA derivatives in detecting PC [18]. The mPHI-2 model demonstrated an ROC analysis with an AUC value of 0.8310, indicating superior diagnostic performance. This model reduced unnecessary biopsies by 56% while maintaining a sensitivity of 78.9% and specificity of 90.3% [19]. The PHI and its modified version, mPHI-2, show promise in improving predictive accuracy but still need further validation in diverse populations.

##### 4Kscore

The 4Kscore test combines total PSA, fPSA, intact PSA, and human kallikrein-related peptidase 2 (hK2), along with clinical information such as age, digital rectal exam (DRE) results, and history of prior negative biopsy, to predict the likelihood of high-grade PC (Gleason score ≥ 7). A meta-analysis by Mi et al. found that 4Kscore has a pooled sensitivity of 0.90 and specificity of 0.44, with an AUC of 0.81, indicating high diagnostic accuracy [20]. In fact, using 4Kscore significantly reduces unnecessary biopsies by distinguishing between patients with a low risk and those with a high risk of clinically significant PC. Decision curve analyses show that 4Kscore effectively minimizes false negatives while reducing biopsy rates, thus providing a more precise and personalized risk assessment in PC detection and management [12]. The 4Kscore test can be influenced by variations in clinical practice and patient demographics, and its moderate specificity indicates room for improvement.

### 2.3. Molecular Biomarkers—DNA/RNA

#### 2.3.1. Somatic Genetic Tests, PTEN/TMPRSS2: ERG

The PTEN/TMPRSS2 assay (Metamark, Cambridge, MA, USA) is designed to predict PC aggressiveness by detecting the presence or absence of the TMPRSS2 fusion gene and PTEN deletion in biopsy samples. PTEN is a tumor suppressor gene, and its inactivation is associated with high Gleason scores and tumor progression [21,22]. The presence of TMPRSS2 fusion and/or PTEN deletion indicates a more aggressive cancer. Although the assay does not strongly correlate with the Gleason score at biopsy, it is unique in its ability to be used in men with atypia or high-grade prostatic intraepithelial neoplasia (HGPIN), potentially leading to earlier diagnosis of aggressive PC [23]. TMPRSS2 rearrangements are less common in transition zone tumors (4%) compared to PTEN deletions (24%), with PTEN inactivation being mutually exclusive with TMPRSS2 rearrangements in stage T1a PC [24]. Additionally, the concurrent presence of TMPRSS2 fusion and PTEN deletion is associated with a poorer prognosis and earlier biochemical recurrence, highlighting the value of these biomarkers in stratifying patient risk and guiding treatment decisions. The absence of both genetic aberrations is associated with a more favorable outcome, aiding prognostic foresight and prediction [23]. The correlation of PTEN/TMPRSS2 assay with clinical outcomes is limited, requiring further studies for prognostic utility refinement.

#### 2.3.2. Combined Prognostic Tests

##### PCA3

PCA3 is a non-coding RNA that is overexpressed in PC tissues and is detectable in urine following a DRE. PCA3 testing offers greater specificity than PSA and does not fluctuate with prostate volume, making it a valuable tool for deciding on repeat biopsies. PCA3 has shown high diagnostic accuracy, especially for patients with previous negative biopsies [25,26]. The Progensa PCA3 test (Gen-Probe Inc., San Diego, CA, USA), approved by the United States Food and Drug Administration (FDA), uses a PCA3 score to guide biopsy decisions. A score below 25 suggests a lower likelihood of a positive biopsy, reducing unnecessary procedures. Studies have demonstrated that PCA3 outperforms fPSA and PHI in predicting biopsy outcomes, with an AUC significantly higher than PSA alone [27]. The high negative predictive value for PCA3 allows for a potentially substantial reduction in unnecessary biopsies, offering a precise and reliable method for assessing PC risk. PCA3 offers greater specificity than PSA but is limited by variability in test performance and the need for standardized collection procedures, with further studies needed in larger and more diverse cohorts.

##### SelectMDx

The SelectMDx (MDXHealth, Irvine, CA, USA) urine test measures biomarker mRNA expression associated with PC. Specifically, it analyzes mRNA levels of the homeobox C6 (HOXC6) and distal-less homeobox 1 (DLX1) genes in urine samples collected after a DRE [28]. In conjunction with clinical risk factors such as PSA density and DRE outcomes, SelectMDx helps to predict the presence of high-grade PC (Gleason score ≥ 7). The use of SelectMDx in clinical practice has demonstrated a high negative predictive value, potentially avoiding 53% of unnecessary biopsies by accurately identifying patients at low risk for clinically significant PC [29]. This study also highlighted that SelectMDx could reduce biopsies by 40.72% in a large cohort, rendering it an effective tool for minimizing invasive procedures and improving the decision-making process for prostate biopsies. However, SelectMDx is not universally accessible and may not be cost-effective in all healthcare settings, with variability in predictive accuracy due to reliance on clinical risk factors [30].

##### ConfirmMDx

ConfirmMDx (MDxHealth, Irvine, CA, USA) is designed as a risk stratification tool for men with a negative prostate biopsy result and aims to reduce the number of repeat biopsies. It detects epigenetic changes by analyzing DNA methylation patterns of tumor suppressor genes such as GSTP1, RASSF1, and APC in prostate tissue samples. Hypermethylation of these genes is linked to PC development [31]. The MATLOC study demonstrated that ConfirmMDx has a sensitivity of 68%, a specificity of 64%, and a negative predictive value (NPV) of 90%, significantly decreasing the number of unnecessary repeat biopsies. The DOCUMENT study confirmed that ConfirmMDx is a significant independent predictor for PC detection in repeat biopsies, with an NPV of 88% [31,32]. ConfirmMDx leverages the epigenetic field effect, where normal cells adjacent to cancer foci exhibit DNA methylation changes, enhancing its predictive power for high-grade cancer. It can reduce unnecessary biopsies by providing a more accurate assessment of cancer risk, especially in cases where traditional diagnostic methods fall short [33]. By analyzing the methylation status of genes like GSTP1, APC, and RASSF1, ConfirmMDx improves the prediction of PC presence in patients with prior negative biopsies [34]. ConfirmMDx offers additional utility by distinguishing between patients who are likely to remain cancer-free and those at risk of developing cancer, thus optimizing patient management and reducing unnecessary invasive procedures [35]. A limitation of ConfirmMDx is the dependence on the accuracy of detecting epigenetic changes, which can vary based on sample quality and field effects.

##### Oncotype Dx

The Oncotype DX assay (Genomic Health, Redwood City, CA, USA) uses fixed, paraffin-embedded prostate needle biopsy tissue samples to predict the aggressiveness of an individual’s tumor. It measures the RNA expression levels of 12 PC-related genes and five reference genes, generating a Genomic Prostate Score (GPS) ranging from 0 to 100. A higher GPS correlates with a higher likelihood of adverse pathology, such as a primary Gleason pattern of 4–5 or non-organ-confined disease. The assay assists in risk stratification and treatment decisions, particularly for clinically low- and low–intermediate-risk patients. Studies have validated its ability to predict adverse pathology and long-term outcomes, with a high GPS being predictive of high-grade and higher-staged disease [36].

Oncotype DX guides safe patient choices for active surveillance over immediate treatment, thereby reducing overtreatment and associated morbidities [37]. The test not only predicts tumor aggressiveness but also helps in determining the appropriate management strategies for patients with localized PC, significantly aiding in decision-making processes [38]. By analyzing the expression of genes involved in androgen signaling, cellular organization, proliferation, and stromal response, Oncotype Dx provides a comprehensive risk assessment that is crucial for personalized patient care [18]. However, OncotypeDX is costly and requires high-quality tissue samples that may necessitate dependence on narrowly available clinical and diagnostic infrastructure.

##### ProMark

ProMark (Metamark, Cambridge, MA, USA) is a quantitative multiplex proteomics-based test that predicts cancer aggressiveness by measuring the levels of eight proteins (DERL1, CUL2, SMAD4, PDSS2, HSPA9, FUS, pS6, and YBOX1) in biopsy specimens. These proteins are involved in cell proliferation, stress response, and signaling pathways. The test provides an individualized risk score for Gleason ≥4 + 3 disease and/or non-organ-confined disease, helping guide treatment decisions. Clinical validation studies have shown that ProMark can predict aggressive cancer and provide valuable prognostic information, assisting in the decision-making process for patients with Gleason 3 + 3 or 3 + 4 disease [39,40]. By utilizing a quantitative multiplex proteomic approach, ProMark can accurately stratify patients with localized PC, aiding in decision-making for active surveillance versus aggressive treatment. Because of these advantages in post-biopsy decision-making, ProMark has experienced an upward trend in utilization since its introduction into commercial selection [41]. The test’s ability to predict non-favorable pathology with a predictive value of 76.9% for scores above 0.80, and favorable pathology with predictive values of 95% and 81.5% for NCCN very low and low-risk groups, respectively, underscores its potential in personalized patient management [42]. A limitation of ProMark is the variability in biopsy sampling and protein expression, which can affect reliability.

##### Prolaris

The Prolaris test (Myriad Genetics, Salt Lake City, UT, USA) quantifies the expression of 31 cell cycle progression (CCP) genes and 15 reference genes to predict tumor aggressiveness and recurrence. The test provides a score that correlates with the risk of disease progression and PC-specific mortality. Measuring progression through the cycle has demonstrated reliability as an independent predictor of PC-specific mortality and biochemical recurrence, aiding in risk-stratification and management decisions for PC patients, substantiated by findings in a cohort of 582 patients where CCP score was associated with both metastatic disease (HR 5.35) and BCR (HR 1.60) [43]. Comprehensive health technology assessments demonstrated the ability of Prolaris to offer prognostic value beyond traditional clinical and pathological factors, averting the incidence of planned or actual treatment for a significant proportion of patients [44]. However, Prolaris is costly and requires high-quality RNA from biopsy samples, necessitating comprehensive health technology assessments for cost-effectiveness.

##### Decipher

The Decipher test (GenomeDx, Vancouver, BC, Canada) is a genomic classifier that measures the RNA expression of 22 genes associated with high-risk PC. It provides a continuous risk score, predicting the 5-year risk of metastasis, 10-year PC-specific mortality, and the likelihood of high-grade disease. Decipher is used to guide post-prostatectomy treatment decisions, including the need for adjuvant radiation therapy or observation. Studies have shown that Decipher scores correlate with biochemical recurrence, metastasis, and PC-specific mortality, offering valuable prognostic information for patients with localized disease. In a recent analysis of prostatectomy tissue from 337 patients, Klein and coauthors reported that 20% of Gleason 3 + 3 prostatectomy specimens had intermediate or high-risk Decipher scores, indicating a potentially more aggressive cancer [45]. Ross et al. demonstrated in a study on 260 patients (99 of which had experienced metastasis) that Decipher scores significantly correlate with biochemical recurrence, metastasis, and PC-specific mortality (*p* < 0.01) [46]. Clinical implementation of Decipher has reclassified 60% of men to lower-risk categories, helping to reduce unnecessary treatments [47,48]. Additionally, Den et al. found that Decipher scores can guide the timing of post-RP radiation therapy in men with high-risk pathology [49]. These findings highlight the role of Decipher testing in optimizing treatment plans and improving patient outcomes through precise risk assessment. A limitation of Decipher is the high cost and the reliance on high-quality tissue samples, which can be limiting and require further validation in broader clinical settings.

##### Apifiny

Apifiny (Armune Bioscience, Kalamazoo, MI, USA) is a blood test that measures the expression of eight PC-specific autoantibodies. It reports a score reflecting the Gleason ≥7 disease risk on biopsy. The test leverages the body’s humoral immune response to cancer, producing autoantibodies against tumor antigens. Apifiny has demonstrated diagnostic value with an AUC of 0.69, sensitivity of 0.603, specificity of 0.69, positive predictive value (PPV) of 0.299, and negative predictive value (NPV) of 0.888, making it a useful tool for assessing PC risk in patients with elevated PSA levels [12]. However, the moderate sensitivity and specificity of Apifiny indicate a need for further improvement with variability introduced by reliance on the humoral immune response.

## 3. Recent Advances in Prostate Cancer Biomarkers: Single-Omics Approaches

Single-omics technologies focus on individual parts of the DNA–RNA–protein central dogma to better understand organ systems and diseases [50]. These technologies provide valuable data with the potential to develop more accurate predictive or prognostic models of a particular condition. However, investigating a single omics data type at a time can make establishing causality difficult.

Our PubMed search of different types of omics revealed the immense utility and potential for the application of single-omics concerning PC (Table 1). In July of 2024, we selected eight types of omics, executed PubMed searches of each term either by itself or in combination with PC, and summed up the number of articles found. The data showed that genomics is the most used technology overall and within PC research. Lipidomics, epigenomics, microbiomics, multi-omics, integrative omics, panomics, metagenomics, and phosphoproteomics have fewer than 500 articles in PubMed related to PC, which reflects their novelty and highlights areas yet to be fully tapped into to further our understanding of PC (Table 1). Our search is by no means comprehensive as there are many types of omics techniques [50]; however, our list focuses on omics techniques we discuss throughout this review and presents some novel techniques for consideration. The raw data used for this analysis are provided in the Supplementary Materials, Tables S3 and S4.

### 3.1. Genomic Biomarkers

Genomic biomarkers correspond to specific genetic properties, including somatic and germline variants, found in the DNA of individuals [51]. These biomarkers can be used in precision oncology to determine the predisposition, prognosis, and treatment response of cancer patients [52]. Genomic biomarkers can be identified based on multiple techniques, mostly through fluorescence in situ hybridization (FISH), genome-wide association studies (GWASs), next-generation sequencing (NGS), and quantitative PCR (qPCR), among others [53]. Although several classical PC genomic biomarkers have been used for detective and prognostic purposes, they do not provide optimal results. Therefore, emerging studies focus on finding novel PC genomic biomarkers that enable a better understanding of PC pathology and enhance treatment approaches. For instance, newly discovered PC risk variants of the *HOXB* genetic locus were found to be hereditary markers of PC predisposition [54]. Additionally, *GOLPH3* and *JUP* genes were upregulated in primary PC tumors compared to adjacent normal tissues [55]. Further genetic aberrations implicated in PC include copy number alterations (CNAs). The overall level of CNAs harbored within PC tumors was found to be a potential clinical marker of prognosis in patients who previously received treatment [56]. A network-based analysis of CNAs of multiple PC cell lines revealed *VGF* as a novel gene biomarker candidate associated with radio-resistance [57]. Furthermore, a study showed that mCRPC patients with two DNA alterations in the *RB1* gene displayed a more aggressive PC phenotype and a decrease in overall survival compared to patients without these alterations. The same study also indicated that poor prognosis is associated with mutations in the genes of the Wnt/β-catenin pathway, where *CTNNB1* was selected as the candidate driving resistance to Enzalutamide [58]. On the other hand, mutations in the Speckle-Type POZ Protein (*SPOP*) gene were shown to be biomarkers linked to better prognosis and enhanced response to androgen deprivation therapy in de novo metastatic castration-sensitive PC, as the loss of function of *SPOP* that happens during early prostate tumorigenesis leads to steady AR expression and upregulated AR signaling [59,60,61].

### 3.2. Transcriptomic Biomarkers

All the information encoded in the genome is expressed through transcription. Transcriptomics refers to the study of an organism’s transcriptome, which encompasses the total number of all RNA transcripts: coding RNA (messenger RNA (mRNA)) and non-coding RNA (ribosomal RNA (rRNA), transfer RNA (tRNA), microRNA (miRNA), small interfering RNA (siRNA), and long non-coding RNA (lncRNA) [62]. Data on RNA transcripts can be gathered either by sequencing technology platforms or nucleic acid hybridization (microarrays) [63]. As such, transcriptomic profiling reveals biomarkers that can be used in precision cancer medicine. A large multi-cohort study that screened the transcriptomic landscape in PC unveiled several novel mRNAs (ABCC5, MPZL1, CCNE1, and RASAL2) as prognostic biomarkers involved in PC progression into metastatic disease [64]. Importantly, Solé et al. sequenced the entire circulating urinary transcriptome and discovered five mRNA biomarkers (FTH1, BRPF1, OSBP, PHC3, and UACA) that are able to differentiate amongst confounding diagnosis of BPH, low-stage PC, and high-stage PC [65]. Moreover, the transcriptomic analysis of normal vs malignant prostate tissue highlighted the role of the non-coding small nucleolar RNA and the small Cajal body-specific RNA (SCARNA22) in the transition of PC from stage T_2c_ to T_3_/T_4._ It also showed Neuronal Regeneration-Related Protein (NREP) transcript to be upregulated in the T_3_ and T_4_ invasive stages of PC, where cancer cells usually metastasize beyond the prostatic capsule [66]. Another metastasis biomarker that can be considered is miR-133a-3p. Indeed, it was shown that miR-133a-3p inhibits the PI3K/Akt pathway, consequently suppressing bone metastasis [67]. A study focusing on the role of two lncRNAs in PC indicated that LINC00261 and LINC00665 are associated with decreased overall survival. Interestingly, both lncRNAs were shown to confer radio-resistance to PC3 PC cells by activating DNA repair mechanisms [68]. Circular RNAs (cirRNAs) are being extensively studied in the field of PC transcriptomics as their production level is correlated with cancer cells’ proliferation and disease progression. In fact, circCSNK1G3 was found to induce PC cancer cells’ growth [69]. Moreover, hsa_circ_0001165 was suggested to promote epithelial-to-mesenchymal transition (EMT) in PC by modulating tumor necrosis factor (TNF) expression [70].

### 3.3. Proteomic Biomarkers

Genomic and transcriptomic modifications can be manifested at the protein level. However, about only 10–20% of all transcriptomic changes result in proteomic ones [71]. Proteins are more challenging to study than nucleic acids as they are prone to post-translational modifications and are susceptible to physiological changes [53]. Proteomic biomarkers can be depicted through mass spectrometry, two-dimensional gel electrophoresis, chromatography, Western blotting, multiplex assays, and protein microarrays [72]. The mapping of proteomes and phosphoproteomes of four PC cell lines uncovered the proteins characterizing the hormonal status and the cancer stage of the cells. For instance, DEAD-Box Helicase 10 (DDX10) was proposed to be a novel marker for cancerous transformation as it was overexpressed in all PC cells, compared to normal prostate epithelial cells. Additionally, Protein Phosphatase 1 Regulatory Subunit 10 (PPP1R10) was shown to be strictly expressed in castration-resistant cells but not in castration-sensitive cells [73]. In a chromatin-directed proteomics study, the SMARCA4 protein was identified as an important modulator affecting a panel of AR target genes, particularly the ones implicated in EMT. The inhibition of this protein also suppressed the growth of VCaP PC cells, which express a castration-resistant phenotype [74]. Furthermore, proteomic profiling of primary prostate tissue cornered Lysyl Oxidase Like 2 (LOXL2) protein as a tumor microenvironment regulator involved in migration and extracellular matrix remodeling [75]. Interestingly, the proteome analysis of PC bone metastatic samples revealed that bone metastases have heterogeneous protein profiles stratified into two phenotypes. The first features aerobic metabolism and high expression of AR targets, while the second is characterized by glycolytic metabolism and overexpression of cell cycle proteins [76]. In addition, Programmed Death-Ligand 1 (PD-L1) protein and cluster of differentiation 8 (CD8) were recently shown to be biomarkers linked with PC progression and poor prognosis in patients with lymph node metastasis after radical prostatectomy [77]. Within the scope of personalized medicine, the Schlafen Family Member-11 (SLFN11) protein was found to be a proteomic biomarker associated with platinum chemotherapy response in patients with castration-resistant PC, where patient-derived PC organoids with SLFN1 knockout did not respond to cisplatin at deadly concentrations [78].

### 3.4. Epigenomic Biomarkers

Epigenomics explores the epigenetic signals on DNA and histones that affect gene expression independently of the original genome sequence. These signatures, including DNA methylation and histone modifications, regulate the dynamic spatial organization of chromatin and influence nucleosome localization, recruitment of transcriptional regulators, chromatin accessibility, and gene expression [79]. Epigenetic dysregulation and the associated molecular variations are classified as constitutive hallmarks of cancer [80,81]. Indeed, the emergence of NGS and other technologies has advanced the understanding of the epigenetic landscape in cancer. Studying these mechanisms has identified potential epi-markers for cancer diagnosis and prognosis [82]. Additionally, anticancer therapies acting against targetable epigenetic markers show potential in pre-clinical and clinical settings, either as single agents or in combination with other therapies [83].

Tolkach et al. identified markedly elevated DNA methylation in the promoter region of the CD24 gene, a key factor in tumor progression, in tissue samples from PC patients compared to those from BPH and normal patients. The study revealed that increased CD24 promoter methylation and the associated high CD24 gene expression are correlated with biochemical recurrence-free survival, tumor grade, and stage in PC patients [84]. In another study, Pu et al. utilized multi-cancer data from The Cancer Genome Atlas (TCGA) and Gene Expression Omnibus (GEO) to identify specific DNA methylation biomarkers with diagnostic value in PC. Through DNA methylation patterns and correlation analysis, they identified eight biomarkers that could distinguish prostate adenocarcinoma (PRAD) at different Gleason stages, five biomarkers that could effectively differentiate PRAD from normal prostate tissues, and six biomarkers that could completely distinguish PRAD from other urinary samples [85]. Moreover, analysis of the DNA methylome in the plasma of patients with localized and metastatic PC revealed that cell-free DNA methylome profiling could reflect clinical outcomes and distinguish between non-metastatic and metastatic PC with high predictive accuracy [86]. Furthermore, histone epi-tags are known to critically influence gene transcription in PC. Wide H3K27me3 histone methylation profiling, using ChIP and 2×400K promoter microarrays, identified several differentially enriched genes in biopsy samples from PC patients compared to normal prostate [87]. Another study by Baratchian et al. further revealed the implication of H3K9me3 in heterochromatin maintenance and progression to anti-androgen resistance in PC. Elevated expression of euchromatic histone methyltransferase 1 (EHMT1), which methylates H3K9, was associated with poor patient outcomes in hormonal therapy [88]. Additionally, deacetylation of H3K18Ac by the Sirtuin 7 (SIRT7) deacetylase is a crucial marker for maintaining key oncogenic properties of PC cells [89]. Immunohistochemical evaluation of SIRT7 expression in malignant and adjacent normal prostate tissues from 57 patients revealed a significant elevation of SIRT7 in tumors and a positive correlation with malignancy grade [90].

### 3.5. Metabolomic Biomarkers

Metabolomics investigates the profile of small bioactive molecules, known as metabolites, which are the final downstream products of gene transcription and protein translation, linking genotype, environment, and phenotype. Metabolite biosignatures provide a unique readout of physiological or disease status and are being utilized as biomarkers to improve specificity and accuracy in patient diagnosis and prognosis [91]. Advances in analytical techniques have enhanced the metabolomic research platform, aiding the study of intricate metabolic reactions and contributing to our understanding of various biological processes and disease mechanisms [91,92]. In cancer, metabolomic profiling is increasingly used for early diagnosis, disease monitoring, and treatment response assessment. Emerging therapies targeting specific metabolic pathways show promise, highlighting metabolomics’ potential in personalized medicine [93,94].

Altered mitochondrial metabolism is suggested to play a role in the development of PC. A study by Franko et al. analyzed the metabolome of prostatic benign and cancerous patient tissues using capillary electrophoresis (CE) and liquid chromatography coupled with mass spectrometry (LC-MS/MS). The results revealed reduced levels of early tricarboxylic acid (TCA) cycle metabolites in PC tissues, whereas the contents of urea cycle metabolites, including aspartate, argininosuccinate, proline, and the oncometabolite fumarate, were higher compared to normal counterparts [95]. Likewise, another study identified five key metabolites (phosphocholine, glutamate, hypoxanthine, arginine, and α-glucose) that could differentiate between PC and benign tissues, as well as between high and low Gleason score samples [96]. Furthermore, a retrospective ex vivo study utilized high-resolution magic angle spinning magnetic resonance spectroscopy (HR-MAS MRS) on tissue samples from 110 radical prostatectomy specimens, including 50 cases with reported PC recurrence. The study evaluated the relationships between metabolites, clinicopathological variables, and recurrence-free survival. The findings showed that higher intratumoral concentrations of spermine and citrate were linked to longer recurrence-free survival. Elevated ratios of (total choline + creatine)/spermine and (total choline + creatine)/citrate were associated with shorter recurrence-free survival [97]. Additionally, plasma and urine metabolites from patients with PC, BPH, and healthy males were examined using LC-MS/MS and gas chromatography–mass spectrometry (GC-MS). Statistical analyses identified several metabolites associated with the urea cycle, TCA cycle, fatty acid metabolism, and the glycine cleavage system that differentiated PC patients from healthy controls with higher sensitivity compared to PSA [98]. A study by Liang et al. examined urine samples from 233 healthy individuals and 236 patients with PC, highlighting the clinical potential of the metabolite 5-hydroxy-L-tryptophan, known for its association with evading the antitumor immune response, as a biomarker for early PC diagnosis [99,100]. Another study explored the associations between the plasma lipidome and clinical outcomes in castration-resistant prostate cancer (CRPC). Lipidomic profiling was conducted on plasma samples from a Phase 1 discovery cohort of 96 CRPC patients, and a prognostic three-lipid signature was identified, consisting of ceramide, sPHIngomyelin, and phosphatidylcholine. This signature was validated in an independent Phase 2 cohort of 63 CRPC patients, where it was further associated with shorter survival, suggesting a potential role of these lipids in disease progression [101].

### 3.6. Microbiomic Biomarkers

The human microbiota is defined as the bionetwork of microorganisms (bacteria, viruses, archaea, and fungi) cohabiting and interacting with the human body in different anatomical sites. A balanced interaction between the host and its microbiota is termed symbiosis, whereas a variation in the number or types of these microorganisms can lead to dysbiosis [102]. The human microbiome refers to the genomic material of all microorganisms inhabiting the human body and is approximately 150 times greater than the complementary human genome count [103]. Therefore, the Human Microbiome Project, an extrapolation of the Human Genome Project, was established to cover the entire genetic content of human and microbial cells found in the human body in order to study the role of microbiota in diseases [104]. The advancements in NGS and genotyping technologies have enabled significant progress in microbiome research and unraveled that only 2% of microbiome diversity is attributed to variability among human genetics [105]. Thus, the human microbiome, also cornered as the second human genome, can be positioned as an impactful complementary approach to conventional human genomics in studying diseases, including cancer.

Microbiomic biomarkers linked to diseases can be assessed based on the types of microorganisms, the metabolite they secrete, and their genetic signatures [106]. As the human microbiome is of a dynamic nature, it is susceptible to alterations linked to diseases. Indeed, research findings correlate infection to increased PC risk through the abundance of pro-inflammatory bacteria and uropathogens, including *Streptococcus anginosus*, *Anaerococcus lactolyticus*, *Anaerococcus obesiensis*, *Actinobaculum schaalii*, *Varibaculum cambriense*, and *Propionimicrobium lymphophilum* in men with positive biopsies for PC [107]. Additionally, profiling of the urinary microbiome, a non-invasive biomarker of PC, using 16S rRNA sequencing revealed that *Faecalibacterium*, *Staphylococcus*, *Ruminococcaceaeu_UCG_002*, *Neisseria*, and *Agathobacter* were the genera with the highest discrepancies when comparing PC groups to normal groups [108].

Pernigoni et al. investigated stool samples of hormone-sensitive vs castration-resistant PC patients to link PC to the gut microbiota. They found that bacteria (such as *Prevotella stercorea*) are more prominent in hormone-sensitive PC patients, whereas *Ruminococcus* and *Bacteroides* species were more abundant in the castration-resistant form of PC [109]. Several studies also suggest that shifts in the microbiome of the prostate and/or other anatomic sites (oral cavity, urinary, and gastrointestinal tracts) result in inflammation, a prominent risk factor of PC [110]. An array-based metagenomic approach showed that *Helicobacteri pylori* were significantly more abundant in 90% of PC tissues compared to normal counterparts. The integration of the *H. pylori*-cytotoxin-associated gene A (CagA) gene within the DNA of PC cells was then validated using PCR and sequencing [111]. The CagA gene is associated with oncogenic pathways, as it activates proto-oncogenes and inactivates tumor suppressor genes [112]. The same study found that three tumorigenic viruses, human cytomegalovirus, human HPV 16, and HPV 18, accounted for 41% of all viruses in PC samples [111]. Importantly, the microbiome can influence the systemic hormonal balance by providing an alternative source of androgens. Indeed, some bacterial species can catabolize androgen and estrogen and metabolize androgen precursors into active androgens, leading to a substantial increase in the levels of these hormones, thereby promoting PC progression from the androgen-sensitive to the castration-resistant stage [109,113].

## 4. Multi-Omics Approaches in Prostate Cancer

### 4.1. Definition and Overview of Multi-Omics

**Figure 1:**
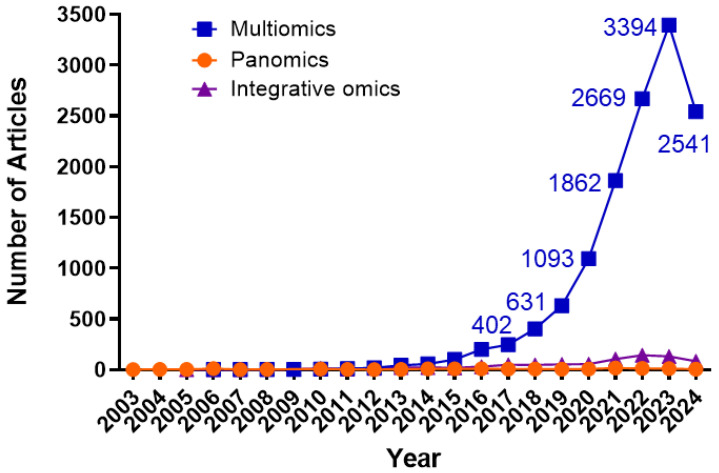
The Utilization of Multi-Omics and Alternative Terms in Research has Grown Exponentially. Line graphs represent the number of peer-reviewed publications in PubMed that contain the terms “Multiomics”, “Panomics”, and “Integrative omics” in PubMed from first use in 2003 until 19 July 2024. Graphs were generated using GraphPad Prism 10.0.00 Software (Boston, MA, USA).

Multi-omics encompasses integrating data from various omics layers—genomics, transcriptomics, proteomics, lipidomics, epigenomics, metabolomics, pharmaco-omics, and microbiomics—to gain a comprehensive understanding of biological systems [114,115,116]. Such integration is vital in revealing complex disease mechanisms like those seen in PC, where the interplay between genetic, transcriptomic, and metabolic changes can significantly influence disease progression and patient outcomes [117]. Due to the significant molecular heterogeneity within the patient population and the molecular variability among individual patients, multi-omics technologies and analyses enable us to gain deep insights into the PC characteristics within each individual, thus supporting precision medicine. A PubMed search of the terms “multi-omics”, “panomics”, and “integrative omics” in July of 2024 revealed that since the first use of these terms in the early 2000s, there has been an explosion of multi-omics utility in research and medicine (Figure 1). Although not used as frequently, multi-omics can be interchangeably referred to as panomics, or integrative omics in the literature (Figure 1). Combined, all three terms have been utilized in over 14,500 PubMed publications. The data show the growing importance of multi-omics in research and medical applications, globally.

We also observed a decline in the number of publications using multi-omics in 2024, despite an overall trend of exponential annual growth. Many factors could contribute to this, including disruptions or global events such as COVID-19, reduced research funding, shifts in research focus, data lags, and publication delays. Despite this decline in the number of publications in 2024, an overall trend of exponential annual growth shows the increasing demand for multi-omics and rapid advancements in omics technologies. The analysis was conducted using GraphPad Prism 10.0.00 for Windows (GraphPad Software, Boston, MA, U.S.A). Raw data from PubMed are provided in the Supplementary Materials, Tables S3 and S4.

Within the past ten years, several pre-clinical studies have shown the importance of integrating multiple data types to obtain a clearer picture of PC [4,118,119,120]. These research papers highlight the use of multi-omics in discovering novel genes, genetic mutations, peptides, metabolites, and potential biomarkers that could be more effective than PSA in diagnosing and surveilling PC [4,118,119,120]. More recent findings also show the successful clinical application of multi-omics in PC prognosis and therapy. For example, Danckaert et al. recently conducted a combined analysis of microbiomics and metabolomics data to study the relationship between radiotherapy and acute gastrointestinal toxicity in PC patients [116]. Wei et al. developed a PC recurrence risk stratification model based on mRNA, miRNA, DNA methylation, copy number variations (CNVs), and lncRNA expression for clinical application [121]. Kiebish et al. identified a panel of serum analytes, which included two proteins, a phospholipid, and a metabolite that complemented pathologic patient features in predicting PC biochemical recurrence [122]. Additionally, combining multi-omics and network pharmacology is becoming more common. Pharmaco-omics can reveal novel drug response mechanisms and guide the application of various drugs in a tailored way for each patient [115,123]. A study in 2009 identified N Methyl glycerine as a novel target for treating PC using pharmaco-metabolomics [124]. Song et al. recently constructed a network of putative PC targets and active chemicals extracted from the plant *Hedyotis diffusa* Willd. In their study, Quercetin and Ursolic acid were identified as the major components involved in PC treatment [125].

### 4.2. Multi-Omics Resources and Tools

Several research groups have developed and/or identified tools, algorithms, and databases for multi-omics analysis that are publicly accessible [126,127,128]. The OMICTools directory (http://omictools.com/) was first compiled in 2014 and listed more than 100 software options and tools related to multi-omics data analysis before it was taken down by its creators in 2021 due to rising maintenance costs [129]. A publicly available, community-maintained list of software packages for multi-omics data analysis has been compiled in GitHub and is regularly updated. Computational algorithms such as iODA and MOGONET are essential for interpreting the vast data generated by omics technologies. Wang et al. leveraged multi-omics data and computational methods to identify six significant biomarkers for early detection of prostate cancer recurrence, demonstrating the multi-omics panel’s effectiveness in identifying high-risk recurrent patients and providing valuable insights into cancer mechanisms [130]. Salachan et al. developed an innovative computational deconvolution method that enabled the isolation of specific gene expression profiles for various tissue components, including stroma, normal glands, prostatic intraepithelial neoplasia (PIN), immune cells, and cancer cells using Spatial Transcriptomics to examine nearly 6750 tissue regions from 12 biopsies of prostate cancer. This approach allowed us to detect gene expression changes during prostate cancer progression and to more accurately differentiate between healthy and diseased areas compared to traditional pathology techniques [131]. Additionally, MiR-452-5p was found to be downregulated in PC through an analysis of data from GEO, ArrayExpress, and the TCGA database. Bioinformatic analysis indicated that the genes targeted by MiR-452-5p are involved in essential processes such as Ras signaling, stem cell pluripotency pathways, and transforming growth factor beta signaling [132]. Also, utilizing TCGA data identified TMED10, TMED2, and SEC31A as positive prognostic biomarkers [133]. Furthermore, artificial intelligence and machine learning can now be used to analyze the many combinations of genomic, proteomic, biochemical, and histological data to predict anticancer drug activity or identify novel drug compounds [134,135]. The algorithms and models used in these and similar projects will be quite useful when applied to PC drug discovery.

Commonly used, publicly available databases for accessing omics data include the National Institutes of Health’s GEO https://www.ncbi.nlm.nih.gov/geo/ and TCGA https://www.cancerimagingarchive.net. Researchers can also access The Cancer Imaging Archive (TCIA) http://cancerimagingarchive.net/ and the Prostate cANcer graDe Assessment Challenge (PANDA) https://www.kaggle.com/c/prostate-cancer-grade-assessment. These datasets provide several molecular characteristics and histomorphology data for PC and other cancers. The websites listed above were last accessed on 21 July 2024. The resources listed here are by no means comprehensive, and this highlights the continued investment in and growth of multi-omics utility in research and medicine. Multi-omics-based analysis is vital to identify and develop potential new biomarkers and improve the clinical management of PC for patients, but proper application requires an understanding of the advantages and limits of such complex analyses.

### 4.3. Advantages of Multi-Omics Approaches in Biomarker Discovery

#### 4.3.1. Molecular Subtyping of Prostate Cancer

The integration of multi-omics data offers several advantages in the discovery and validation of biomarkers for PC. By analyzing data across multiple biological layers, multi-omics allows for a more detailed understanding of disease mechanisms, overcoming the limitations of single-omics studies. Multi-omics facilitates the identification of more accurate and clinically relevant biomarkers. The prediction of disease progression can be enhanced using multi-omics by integrating diverse datasets. This enables the development of personalized medicine by identifying unique molecular profiles that predict individual responses to specific therapies, ensuring that patients receive the most effective treatments based on their omics profiles.

As part of The Cancer Genome Atlas Study, analysis of 333 primary prostate carcinoma cases showed that 74% belong to subtypes having specific gene fusions (*ETV1/4, FLI1,* and *ERG*) or mutations (*FOXA1, IDH1,* and *SPOP*). As for epigenetic profiles, heterogeneity was found, including an *IDH1*-mutant subset with a methylator phenotype. Also, the activity of AR varied based on a subtype-specific manner, where the highest levels of AR-induced transcripts were associated with *SPOP* and *FOXA1* mutant tumors [136].

#### 4.3.2. Prognostic Biomarker Discovery

Multi-omics integrates data from various biological layers to offer a comprehensive perspective on cellular processes. This approach aids in identifying reliable and robust biomarkers by capturing the complex nature of biological systems, thereby enhancing the sensitivity and specificity of biomarker discovery. By reducing noise and amplifying signals from relevant biological features, multi-omics enables more accurate identification of disease states and potential therapeutic targets [137].

#### 4.3.3. Single-Cell Multi-Omics

Single-cell omics technologies, such as single-cell RNA sequencing (scRNA-seq), single-cell DNA sequencing (scDNA-seq), and single-cell proteomics, have significantly advanced PC research by providing detailed insights into cellular heterogeneity and the tumor microenvironment (TME) [138]. For instance, Saha et al. identified 389 cell-enriched lncRNAs in PC cells and the TME, which were associated with regulatory elements and exhibited changes during prostate cancer progression. PC-specific lncRNAs correlated with AR mutations and treatment responses, while TME-specific lncRNAs were linked to RB1 gene deletions and poor prognosis, highlighting their potential as biomarkers for disease outcomes [139]. Ren et al., through genome-wide sequencing and transcriptomic analysis of samples from 65 untreated PC patients, discovered a high frequency of CHD1 deletions in Chinese patients, associated with more mutations in the AR upstream activator gene and a low TMPRSS2-ERG fusion rate. They also identified PCDH9 as a critical tumor suppressor gene and PLXNA1 as a gene whose amplification predicts metastasis and poor survival [119]. Wang et al. developed the Prostate Cancer Model Repository (PCMR) with primary patient-derived cells, revealing distinct genetic backgrounds and cell proliferation mechanisms between tumor and benign samples and identifying AGR2 as a prognostic indicator and crizotinib as a selective drug for PC primary cells [140].

#### 4.3.4. Spatial Multi-Omics

De Vargas Roditi et al. used single-cell mass cytometry to explore proteomic heterogeneity within localized prostate cancer, identifying rare cell subpopulations and distinct immune, stromal, and epithelial cell phenotypes associated with high-grade PC [141]. Additionally, scRNA-seq was employed to analyze metabolic features in the TME, discovering that epithelial cells are more metabolically active and plastic than stromal cells, with neuroendocrine (NE) cells exhibiting high metabolic rates, which may explain the aggressiveness of NE prostate cancer [137]. Bian et al. utilized scRNA-seq, spatial transcriptomics, and bulk ATAC-seq to identify a subset of club cells characterized by high SOX9 and low AR expression, suggesting these cells play a significant role in PC progression [142]. In summary, the integration of multi-omics offers a thorough understanding of PC by combining data from multiple biological layers. These advancements collectively promise to improve diagnostic accuracy and therapeutic strategies in PC.

### 4.4. Clinical Applications

#### 4.4.1. Personalized Therapy

Multi-omics research provides new avenues for personalized treatments of cancers and serves as a link between molecular markers and predictors of therapeutic responses. Specifically, biomarker-specific treatments that target the biological traits of a specific tumor are the future of precision medicine [143]. In terms of PC, Murphy et al. were the first to integrate multi-omics data based on DNA methylation, coding and non-coding transcripts, proteins, and glycosylation with associated clinical data to develop a single panel for classifying PC aggressiveness [144]. Wang et al. also developed models to integrate three omics, gene expressions, methylation-related gene expressions, and miRNA to aid in the prediction of the risk of recurrence of PC post-radical prostatectomy. Consequently, *TELO2*, *ZMYND19*, *miR-143*, *miR-378a*, cg00687383 (*MED4*), and cg02318866 (*JMJD6*; *METTL23*) were identified as biomarkers associated with the recurrence risk, implying their prognostic predictive value [145]. Another study pursued the same prognostic goal but incorporated proteomics, metabolomics, and lipidomics. A serum-based panel of four biomarkers (TNC, 1-MA, APOA-IV, and PA18:0–22:0), in combination with pathologic Gleason score and tumor stage, were found efficient in predicting disease prognosis, specifically in terms of PC recurrence post-radical prostatectomy [146].

Zheng et al. assessed metastatic potential via stemness in PC by narrowing analyses to 18 signaling and stemness-associated pathways. PC patients classified as “high stemness” with the highest malignancy and tumor mutation load, worst prognosis, and immunosuppression were found to be sensitive to androgen deprivation therapy, taxanes, and immunotherapy. PC patients classified as “low stemness” with the lowest malignancy and tumor mutation load, best prognosis, and highest immune infiltration were found to be sensitive to platinum-based chemotherapy yet resistant to immunotherapy. Interestingly, these “high”, “medium”, and “low” stemness subtypes were conserved across the pan-tumor landscape, and a nine-gene stemness subtype predictor with a comparable capability to the 18 signaling pathways was developed to diagnose cancer and predict treatment response and outcome characteristics [145].

In terms of immune response, a multi-omics study by Zhu et al. verified the influence of macrophages on PC development and progression by identifying 307 MRMGs. Nine MRMGPS-based genes (*CTSZ*, *FCGRT*, *GOLM1*, *SMIM22*, *ACPP*, *FAM3B*, *TFF3*, *PCA3*, and *MSMB*) were found to be associated with PC prognosis and prediction of treatment response with associations linked to tumor microenvironment [146]. Another multi-omics-related study concluded that expression of enhancer of zeste homolog 2 (EZH2), which is associated with DNA methylation modifications, tumor mutation burden, tumor neoantigen burden, mismatch repair, tumor microenvironment, and genes related to the immune system, served as an independent predictor of prognosis and immune response in PC patients [147].

A multi-omics approach in PC clinical trials has recently emerged. As of September 2024, there are seven trials listed at www.clinicaltrials.gov (accessed on 2 September 2024) with four studies currently recruiting study participants. Recruiting sites are based in Singapore, Spain, Canada, and Taiwan. Disease stages under investigation range from localized primary disease to mCRPC. No studies have yet been completed, but disseminated results in the future will better inform clinical options for PC patients.

#### 4.4.2. Role of Artificial Intelligence (AI) and Machine Learning (ML)

AI, including machine learning (ML) techniques, has the potential to empower the role of multi-omics in improving the management of cancer cases, namely through cancer subtyping, risk assessment, prognostication, prediction, and decision-making [148]. For instance, a multi-omics-based study identified a prognostic risk score model comprising six copy number variant-driven lncRNAs. Among the six lncRNAs (CCAT2, LINC01593, LINC00276, GACAT2, LINC00457, and LINC01343), GACAT2 exhibited the most unfavorable recurrence-free survival outcome, while LINC01593 had the most favorable rate (*p* = 0.034). Based on the pattern of the six lncRNAs, a recurrence predictive model (*p* = 0.024) was developed [149].

### 4.5. Challenges and Future Directions in Clinical Translation

Multi-omics holds great promise in identifying diagnostic and prognostic biomarkers to increase the availability of effective treatments and improve survival outcomes. However, this field of study is new and has several challenges yet to overcome. One of the main difficulties related to the use of multi-omics in the diagnostic and therapeutic sectors of PC is tumor heterogeneity, which makes it challenging to develop biomarkers that accurately reflect the characteristics of the entire tumor [6]. Another challenge relates to biomarker specificity for PC [150]. Many studies have not considered the type or stage of PC in data analyses. The large amounts of heterogeneous data in PC biology, diverse data formats, dynamic ranges, and sources of analytical and experimental errors create hindrances to integrative approaches for this disease [151].

## 5. Conclusions

While traditional biomarkers for prostate cancer are well established and commonly used for clinical screening, there is a significant need for more specific biomarkers to enhance diagnosis and prognosis accuracy. This need is particularly acute for high-risk populations, who are often diagnosed at advanced stages of the disease, where curative options are limited. The integration of multi-omics approaches to simultaneously identify biomarkers at the genomic, transcriptomic, proteomic, metabolomic, and epigenomic levels provides a robust foundation for developing effective PC therapeutics tailored to these biomarkers. This comprehensive approach enhances drug discovery and translational research and the use in cancer research overall has grown exponentially. As multi-omics tools are increasingly utilized in both research and clinical settings, they will advance the field of personalized treatments and precision medicine for PC, ultimately improving patient outcomes.

## Figures and Tables

**Table 1:** PubMed omics search terms and their relative results from 1951 to 2024.

Omics Approach	Number of Publications in Cancer	Number of Publications in Prostate Cancer	Years of Implementation
Genomics	424,352	17,357	1951–2024
Transcriptomics	99.833	4899	1982–2024
Proteomics	44,766	1995	1996–2024
Microbiomics	19,232	364	1979–2024
Metabolomics	16,066	793	2000–2024
Epigenomics	11,053	477	1975–2024
Multi-omics	5782	158	2009–2024
Integrative Omics	5582	174	2007–2024
Panomics	4870	147	2005–2024
Metagenomics	2333	35	2006–2024
Lipidomics	2173	120	2003–2024
Phosphoproteomics	2057	73	2001–2024
Glycoproteomics	703	58	2005–2024

## Data Availability

The data supporting the conclusions of this review article will be made available by the authors upon request.

## Supplementary Materials

The following supporting information can be downloaded at https://www.mdpi.com/article/10.3390/biology13100762/s1, Table S1: PubMed timeline results by year search query: genomics; Table S2: PubMed timeline results by year search query: genomics + “prostate cancer”; Table S3: PubMed timeline results by year search query: “multi-omics”; Table S4: Raw data from PubMed.
